# Implementation and sustainability of hospital-based addiction consultation services

**DOI:** 10.1186/s13722-026-00695-x

**Published:** 2026-07-10

**Authors:** Sandra K. Evans, Allison J. Ober, Allyson Gittens, Alex Peltz, Itai Danovitch

**Affiliations:** 1 RAND Corporation, 1776 Main St., Santa Monica, CA 90407-2138 USA; 2 Department of Psychiatry & Behavioral Neurosciences, Cedars-Sinai, 8700 Beverly Blvd, Los Angeles, CA 90048 USA

**Keywords:** Addiction Consultation Services (ACS), Opioid Use Disorder (OUD), Medication for Opioid Use Disorder (MOUD), Substance Use Disorder (SUD), Hospital-based care, Sustainability, Program Sustainability Assessment Tool (PSAT), Treatment linkage, Implementation barriers

## Abstract

**Background:**

Substance use disorder (SUD) is prevalent among hospitalized patients, yet most acute care settings do not routinely provide evidence-based SUD treatment. Hospital-based addiction consultation services (ACSs) have emerged as a promising strategy to increase the use of medications for SUD and improve linkage to post-discharge care. However, despite evidence supporting ACS effectiveness, these programs remain difficult to sustain, and their growth has been concentrated in a relatively small minority of hospitals. Understanding the factors that enable ACS sustainment and the barriers programs encounter is critical to expanding evidence-based SUD treatment across the broader hospital landscape.

**Methods:**

Using a mixed methods approach, we conducted semi-structured interviews and surveys with 21 leaders and representatives of ACSs at 17 hospitals across the United States. Participants were identified through purposive and snowball sampling via addiction medicine professional networks and correspondence. Interviews explored reasons for ACS initiation, configuration, barriers, and factors supporting sustainment, guided by constructs from the Program Sustainability Assessment Tool (PSAT), including environmental support, funding stability, organizational capacity, strategic planning, partnerships, communications, monitoring, and evaluation. Survey items based on the PSAT asked participants to rate the extent to which their hospital had engaged in various activities related to implementation and sustainment of the ACS. We describe common themes based on qualitative coding of interview data and the analysis of median scores and interquartile ranges for each relevant PSAT domain.

**Conclusion:**

Findings suggest that funding stability and lack of adequate post-discharge treatment services are ongoing barriers to ACS sustainability. Most participating ACSs are supported by diversified funding and cross-disciplinary staffing, but fee-for-service models can limit funding for multidisciplinary roles. While high median survey scores across most PSAT domains reflect strong support for ACS sustainment, addressing financing barriers is crucial for long-term stability of ACSs in sustaining care for hospital inpatients with SUDs.

**Trial registration:**

Clinical trial number: not applicable.

**Supplementary Information:**

The online version contains supplementary material available at https://doi.org/10.1186/s13722-026-00695-x.

## Introduction

In 2024, 16.8% of the U.S. population (48.4 million people) had a substance use disorder (SUD), including 27.9 million with an alcohol use disorder and 28.2 million with any drug use disorder; of those with a drug use disorder, 4.9 million had an opioid use disorder (OUD) [1]. People with SUDs frequently present to acute care settings with direct and indirect consequences of their substance use, and experience markedly worse outcomes than the general population [2]. People with an SUD have a 24% greater likelihood of an unplanned readmission to the hospital [3] and people with OUD are four times more likely to have a fatal overdose in the first two days after hospital discharge [4], indicating the need for additional support both during and after the hospital stay.

Addiction consultation services (ACSs) can improve the implementation of evidence-based treatments (such as medications for alcohol or opioid use disorder) during acute hospitalization and facilitate post-discharge treatment engagement for people with SUD [5–7]. For people with OUD in particular, the inpatient setting represents a pivotal touchpoint where patients can initiate life-saving medications for OUD and receive facilitated linkage to follow-up care in the community.

Results from the Substance Use Treatment and Recovery Team (START) randomized clinical trial (RCT)—a hospital-based addiction consultation service intervention that provides proactive OUD identification, MOUD initiation, and discharge planning/linkage support delivered by an interprofessional team—showed that, compared with usual care, START participants were significantly more likely to initiate MOUD during hospitalization, have an OUD-focused discharge plan, and initiate or continue MOUD after discharge [5]. Other studies such as a pilot RCT of START, as well as a stepped-wedge trial and multiple observational studies also have demonstrated that ACSs improve linkage to MOUD and post-discharge care, and have found ACS implementation to be associated with reduced rates of patient-directed discharges, reduced in-hospital stigma, improved patient trust in hospital clinicians, and an overall reduction in post-discharge mortality [6–10].

ACSs are generally comprised of an interprofessional team capable of delivering MOUD and supportive services [11]. The most common roles are a physician to medically manage SUD and a care manager to deliver brief interventions and coordinate linkage to aftercare [5, 8, 11]. Other roles and ACS activities include social workers to provide psychosocial assessments and assist with care planning, nurses to help administer medication and monitor withdrawal, pharmacists to guide safe medication use, and peer recovery coaches to offer lived-experience support.

ACS teams also frequently provide education and support to hospital staff to improve competence in SUD management, reduce stigma, and model best practices for addiction treatment. Activities can include withdrawal management, pain management, harm reduction counseling, and direct coordination of transitions of care to outpatient SUD treatment or community-based resources, as well as follow-up phone calls. The composition and focus of ACS teams vary according to institutional needs and resources [8]. Team composition and department location vary, and specific placement depends on institutional resources, size, and priorities, though an addiction medicine or psychiatry physician is generally considered essential [8].

Despite growing evidence supporting the effectiveness of ACSs, there is limited information on their implementation and sustainment, with just one article to date (Priest et al., 2020) addressing implementation issues [12]. Based on interviews with addiction physicians at 16 U.S. hospitals involved with or planning ACSs, Priest et al. suggest that barriers to the development and operations of ACSs include stigma and discrimination from both hospital administration and external stakeholders, limited addiction-related resources, challenges with access to community-based treatment programs, restrictive MOUD policies, and challenges stemming from low-reimbursement models [12]. There remains a substantial gap in the literature regarding factors that support initial implementation and long-term sustainability, financing, and scale-up of ACS programs across diverse hospital environments. This information is critically needed to advance policy support and funding for, and dissemination of, ACS programs in hospitals with varied resources and capabilities.

To address this gap, we used a convergent parallel mixed-methods study approach with web-based surveys and semi-structured qualitative interviews with 21 addiction medicine professionals working in ACSs in 17 hospitals across the U.S. to understand reasons ACSs were initiated, how they were initially configured and have evolved over time, and key factors supporting their initiation and sustainment. This methodological approach allowed us to explore relatively comprehensive data and to compare results of qualitative and quantitative data. In this article, we present findings from this work and offer considerations for hospitals interested in implementing and sustaining an ACS to improve evidence-based care for hospitalized patients with OUD.

## Methods

Using a convergent parallel mixed-methods design, we combined semi-structured interviews and surveys to gather insights from leaders and representatives of ACSs at hospitals throughout the U.S. In interviews, researchers asked interviewees about the background of each ACS including how it started and how it has been sustained over time.

Our survey and qualitative work were guided by key sustainability constructs highlighted in the Program Sustainability Assessment Tool (PSAT) [13]. The PSAT was developed to guide assessment of capacity for sustainment of public health programs.

### Participants

We used a purposive sampling approach to select participants. Criteria for selection included a focus on leaders of ACSs in hospitals around the U.S. Specifically, we sought out hospital administrators or other staff who were knowledgeable about the implementation of an ACS in a hospital setting.

We contacted potential participants through mass emails to widely used listservs including the Academy of Consultation Liaison Psychiatry Addiction Consult Service interest group, and the American Society of Addiction Medicine listserv of hospital addiction medicine specialists. We followed up to schedule interviews with those who responded. We also used snowball sampling to locate additional representatives of ACSs. Finally, we developed a list of names of hospital leaders and others affiliated with ACSs based on academic journal publications. In some instances, we cold-emailed contacts from that list. In other instances, the names on that list overlapped with contacts from listserv emails. We conducted interviews until we reached the saturation point when all three researchers agreed that very similar topics and themes were emerging from each interview. Interviewees were asked to complete a survey after each interview was completed.

### Procedures

We conducted semi-structured interviews with 21 leaders and individuals representing ACSs at 17 hospital sites. These interviews took place between August and December 2024. Interviews lasted between 40 and 60 min and were conducted virtually via Microsoft Teams. Audio from interviews was transcribed to facilitate analysis.

We developed a semi-structured interview guide consisting of open-ended questions designed around PSAT sustainability factors including environmental support, funding and funding sustainability, organizational capacity, program evaluation and adaptation, communications, strategic planning and implementation strategies, and partnerships (within and outside the hospital setting). The guide also included questions on barriers related to implementing and sustaining the ACS (see Supplementary Table S1).

After each interview, we emailed a link to an online questionnaire to each participant. Survey items asked participants to rate several factors related to the ACS: environmental support, funding stability, strategic planning and organizational capacity, the extent of monitoring and evaluation, and external partnerships.

**Fig. 1:**
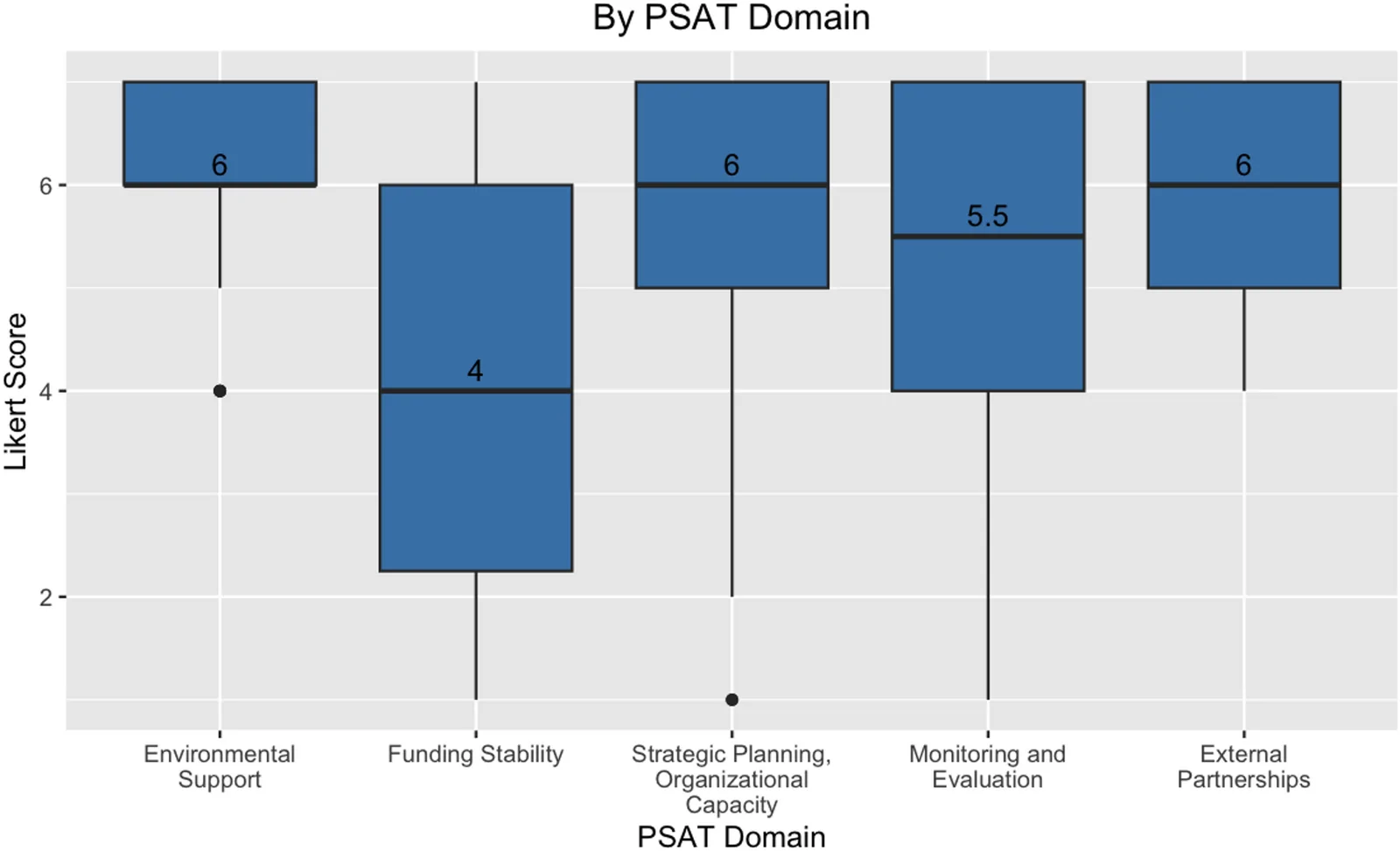
This figure shows median and IQR results from the survey data, organized by the PSAT domains: environmental support, funding stability, strategic planning and organizational capacity, monitoring and evaluation, and external partnerships. High median scores (above 5 on a scale from 1 to 7) indicate strong support of each sustainability factor

Survey responses were aggregated at the domain level (see Fig. 1) by combining scores from each category. The reliability of these combined scores was assessed using Cronbach’s alpha to assess the internal consistency of the constructs, with the following results: 0.79 for environmental support, 0.83 for funding stability, not scored for external partnerships, as there was only one question, 0.90 for monitoring and evaluation, and 0.79 for strategic planning and organizational capacity. Strategic planning and organizational capacity were consolidated into a single domain due to highly correlated survey items as both domains consider the roles and abilities of ACS staff. Participants rated statements in each domain on a scale from 1 to 7, indicating the extent to which the hospital had done the stated activity, toward implementation and sustainment of the ACS, with 1 indicating “no extent” and 7 indicating “to a very great extent.”

Through the integration of both interview and survey data, this study was able to collect comprehensive data about each ACS. Participants were offered $100 in the form of a gift card as an incentive to complete the interview and questionnaire.

### Qualitative interview data analysis

To analyze interview data, we used an inductive and deductive approach. First, we developed a codebook derived from the question topics in the interview guide in PSAT categories (see Supplementary Table S2). After reading all transcripts, we developed codes around emergent themes. Codes were used for initial categorization and theme identification. Three researchers then practice-coded and discussed the codebook at length to refine and finalize the codebook. After the codebook was finalized, the researchers completed an inter-rater reliability assessment.

To assess inter-rater reliability, the coding team (the PI and two team members) conducted three rounds of coding. First, they coded the same transcript to help refine the codebook and add to the code definitions. Next, each researcher coded a unique transcript within Dedoose, a qualitative analysis software program [14]. All coded excerpts were then exported to Excel; the two other coders who had not coded in Dedoose then blind-coded the excerpts in Excel. The purpose of this exercise was to test the extent to which coders applied the same codes to each excerpt across three different transcripts. The results were three transcripts triple-coded by the three researchers. Next, we conducted an IRR test (Krippendorff’s alpha). We used Krippendorff’s alpha because it is appropriate for evaluating agreement among three coders on categorical qualitative codes [15]. The results were lower than optimal for intercoder reliability, so we reviewed discrepancies and clarified codes in the codebook. We then repeated the assessment with a revised codebook. The Krippendorff’s alpha test on main (parent) codes equaled 0.704 (95% CI: 0.582, 0.813), which is considered acceptable [15].

After coding was complete, we reviewed candidate themes and iteratively refined them. When new themes emerged, the team discussed whether they represented a coherent pattern of meaning relevant to the research question and whether to retain them [16]. To support analytic rigor, themes generally reflected patterns expressed by multiple participants; however, themes were also retained when they were substantively important to the research question despite lower frequency.

### Survey data analysis

We summarized central tendencies using median scores and interquartile ranges for each relevant PSAT domain (Environmental Support, Funding, Organizational Capacity, Partnerships, Adaptability and Strategic Planning, Communications, and Program Evaluation). We created box-and-whisker plots for each domain, using the ggplot2 package in R [17]. Each box-and-whisker plot represents the distribution of the score for each statement for all respondents across a range from 1 to 7. Each box shows scores from the 25th to the 75th percentile, with the median shown as a line across the middle of the box. The whiskers extend from the box to the smallest and largest scores that are not considered outliers. Scores outside these fences (the lower fence is set at 1.5 times the interquartile range below the 25th percentile, and the upper fence is 1.5 times the interquartile range above the 75th percentile) are considered outliers and are shown as individual dots.

## Results

### Participant characteristics

Of the 21 interviewees, 18 (86%) responded to the brief demographic and sustainability survey. Of these, most (83%) were physicians, female (61%), and White (83%); 22% were Hispanic or Latino. The majority (89%) of interviewees who responded to the survey were currently working as a clinician on the ACS and most (61%) ACSs had been implemented for longer than five years (see Table 1 for additional demographic data). Because the survey was designed to collect quantitative items from interview participants, the number of survey respondents was necessarily limited. Therefore the analysis of survey data is descriptive and used to provide additional insight and triangulation with the qualitative findings. Additionally, the number of people involved in each ACS was relatively small so when quoting participants, we refer to generic ID numbers to protect participants’ confidentiality.

**Table 1:** Participant demographics

Participants	*N*	(%)
***N*** ** (Total Interviewees)**	**21**	
***N*** ** (Survey Respondents)**	**18**	**(86%)**
**Participant’s Role at Hospital**		
Physician	15	(83%)
Administrator	3	(17%)
**Gender**		
Female	11	(61%)
Male	7	(39%)
**Hispanic/Latino**	**4**	**(22%)**
**Race**		
American Indian/Alaskan Native	0	
Asian	2	11%
Black/African American	0	
Native Hawaiian/Pacific Islander	0	
White	15	83%
Other Race	1	6%
**Participant is currently a clinician on an ACS**	16	(89%)
**Participant’s tenure as clinician on ACS**		
Less than a year	2	(11%)
1–2 Years	2	(11%)
3–5 Years	2	(11%)
Longer than 5 Years	10	(56%)
**Tenure of ACS**		
Less than a year	2	(11%)
1–2 Years	2	(11%)
3–5 Years	3	(17%)
Longer than 5 Years	11	(61%)

### About participating hospitals

Participating hospitals (*N* = 17) were located across four major geographic regions in the United States, with seven in the Northeast, five in the West, three in the Midwest, and one in the South. Most hospitals’ ACSs were established within the past decade, with six sites reporting a tenure of 10 or more years, seven reporting 5–9 years, and four reporting 1–4 years since inception. With respect to placement within the hospital, ACSs mostly resided within Psychiatry (*n* = 6) or Addiction Medicine departments (*n* = 4), while others were embedded in general medicine (*n* = 2), hospital medicine (*n* = 1), or internal/family medicine (*n* = 2); one hospital noted a combined psychiatry/VA placement.

Most hospitals (*n* = 10) utilized an interprofessional ACS model [11]. Another five sites described a psychiatry consultation-liaison service model, while two sites reported practice-based or individual consultant approaches.^1^ ACS cross-departmental staffing included various combinations of addiction medicine physicians, nurse practitioners, social workers, and frequently incorporated medical trainees (fellows, residents, medical students), peer recovery coaches, and, in some programs, addiction counselors, care managers, patient navigators, or researchers. Initial program funding was most commonly derived from hospital or health system sources (*n* = 10), followed by grant funding (*n* = 7) and, to a lesser extent, government (non-grant), philanthropic/donor, volunteer/unfunded, or other sources. Current funding was diversified across hospital/health system support (*n* = 13), professional billing revenue (*n* = 3), grant (*n* = 5), and government (non-grant) funding streams (*n* = 4; see Table 2).

**Table 2:** Characteristics of ACSs

Org #	Region	Tenure of ACS	Home of ACS in Hospitals	ACS Category	Mentioned in Englander taxonomy?	Types of Staff	Initial Funding - Standardized	Current Funding - Standardized
1	Northeast	10 or more years ago	Psychiatry	Addiction Consult Model/Psychiatry consult liaison service	Yes	Addiction Physicians, Attending, Psychiatry Residents, Social Workers	Hospital/Health System Funding	Hospital/Health System Funding; Philanthropic/Donor Funding; Grant Funding; Government Funding (Non-Grant)
2	Northeast	10 or more years ago	Addiction Medicine	Addiction Consult Model/Interprofessional addiction consult service	Yes	Addiction Medicine Fellow, Attending Physician, Nurse Practitioners, Peer Coach, Residents & Students, Social Worker	Hospital/Health System Funding	Professional Billing Revenue; Hospital/Health System Fundin
3	Mid-Atlantic	5–9 years ago	Addiction Medicine	Practice-based model/Hospital-based opioid treatment (HBOT)	Yes	Addiction Medicine Fellows, Attending, Peers, Physicians, Social Workers	Grant Funding	Hospital/Health System Funding
4	Northeast	5–9 years ago	Addiction Medicine	Addiction Consult Model/Interprofessional addiction consult service	Yes	Addiction Medicine Fellows, Addiction Medicine Physicians, Nurse Practitioners, Resource Specialists, Social Works	Hospital/Health System Funding; Grant Funding	Professional Billing Revenue; Grant Funding
5	Northeast	1–4 years ago	Psychiatry	Addiction Consult Model/Psychiatry consult liaison service	Yes	Addiction Medicine Fellows, Physicians, Psychiatrists, Social Worker	Hospital/Health System Funding	Hospital/Health System Funding; Other/Not Specified
6	Midwest	1–4 years ago	Psychiatry	Addiction Consult Model/Psychiatry consult liaison service	No	Interns, Psychiatry Fellows, Psychiatrists, Residents, Social Worker	Hospital/Health System Funding	Hospital/Health System Funding; Other/Not Specified
7	Northeast	5–9 years ago	Psychiatry	Addiction Consult Model/Psychiatry consult liaison service	No	Addiction Counselor, Addiction Medicine Fellows, Peer, Physicians or Nurse Practitioner, Social Workers	Grant Funding; Government Funding (Non-Grant)	Hospital/Health System Funding
8	West	5–9 years ago	Psychiatry	Addiction Consult Model/Interprofessional addiction consult service	Yes	Attending Addiction Physician, Attending Physicians from Internal and Family Medicine, Nurse Practitioners, Social Workers	Grant Funding; Philanthropic/Donor Funding; Government Funding (Non-Grant)	Hospital/Health System Funding; Government Funding (Non-Grant)
9	West	5–9 years ago	Internal and Family Medicine	Practice-based model/Hospital-based alcohol treatment	Yes	Attending Physicians from multiple departments, Nurse Practitioners, Patient Navigators, Social Workers	Grant Funding; Philanthropic/Donor Funding; Government Funding (Non-Grant)	Hospital/Health System Funding
10	West	1–4 years ago	Medicine	Addiction Consult Model/Psychiatry consult liaison service	Yes	Attending Physicians, Fellows from Addiction Medicine Psychiatry Toxicology, Medical Students, Residents, Scheduler, Social Worker	Grant Funding	Hospital/Health System Funding; Grant Funding
11	West	5–9 years ago	Hospital Medicine	Addiction Consult Model/Interprofessional addiction consult service	Yes	Addiction or Hospital Medicine Physicians, Care Coordinator, Fellows, Nurse Practitioners, Social Worker	Grant Funding; Government Funding (Non-Grant)	Hospital/Health System Funding
12	South	10 or more years ago		Addiction Consult Model/Interprofessional addiction consult service	Yes	Addiction Councilor, Care Managers, Nurse Practitioners, Peers, Physicians	Other/Not Specified; Hospital/Health System Funding?	Government Funding (Non-Grant); Hospital/Health System Funding
13	Midwest	10 or more years ago		Addiction Consult Model/Individual consultant	Yes	Addiction Medicine Physicians, Councilors, Social Workers	Volunteer/Unfunded; Other/Not Specified	Hospital/Health System Funding
14	Northeast	5–9 years ago	Medicine	Addiction Consult Model/Interprofessional addiction consult service*	No	Attending Physicians, Fellows, Medical Trainees, Nurse Practitioner, Residents, Social Workers, Students, Peer	Hospital/Health System Funding	Hospital/Health System Funding; Grant Funding
15	West	5–9 years ago	Psychiatry and VA affiliation	Addiction Consult Model/Psychiatry consult liaison service*	No	Addiction Fellows, Addiction Psychiatrics, Residents in General Medicine and Psychiatry, Social Workers, Students	Volunteer/Unfunded	Hospital/Health System Funding; Professional Billing Revenue
16	Northeast	1–4 years ago		Addiction Consult Model/Interprofessional addiction consult service*	No	Fellows, Hospitalists, Peer, Researcher	Other/Not Specified	Other/Not Specified
17	Northeast	5–9 years ago	Internal Medicine	Addiction Consult Model/Interprofessional addiction consult service	Yes	APPs, Addiction Medicine Physicians, Health Promotion Advocates, Fellows, Social Workers, Social Worker	Hospital/Health System Funding; Grant Funding	Hospital/Health System Funding

### Themes about initiating and sustaining ACSs

In this section, we present themes that emerged from interview and survey data regarding the factors participants deemed important for initiating and sustaining ACSs over time. Results are organized according to the PSAT categories: Environmental Support, Funding, Organizational Capacity, Partnerships, Adaptability and Strategic Planning, Communications, and Program Evaluation. We also include a category addressing cross-cutting barriers to implementing and sustaining the ACS, which may span multiple PSAT domains and are not limited to a single domain.

Survey findings show that overall, median survey scores within PSAT domains were high, with most above 5 (on a scale from 1 to 7), indicating fairly strong support of each factor toward sustainability of the ACS. The funding stability score was lowest. Figure 1 provides an overview of survey responses in each PSAT domain.

Table 3 provides a summary of themes based on qualitative interview data. We describe themes and survey responses in greater detail below.

**Table 3:** Overview of themes from qualitative interviews including themes related to PSAT domains and cross-cutting barriers

PSAT Domain	Theme
Domain 1: Environmental Support (Including internal partnerships)	1. Widespread stakeholder buy-in reflected shared recognition of unmet inpatient OUD needs. 2. Leadership support and champions across levels were central to initiation and sustainment.
Domain 2: Funding Stability	3. Start-up support enabled ACS launch, followed by efforts to transition to more durable institutional support. 4. Funding remained structurally precarious despite diversification.
Domain 3: Strategic Planning, Organizational Capacity	5. Cross-disciplinary relationships within the hospital expanded reach and supported sustainment. 6. Interprofessional staffing and cross-departmental participation strengthened organizational capacity.
Domain 4: Monitoring and Evaluation	7. Monitoring and evaluation were viewed as essential, but measurement approaches were not required and were spontaneously developed. 8. Sites used different metrics to demonstrate value, and some commonly used hospital metrics were viewed as a poor fit.
Domain 5: External Partnerships	9. External partnerships were essential for post-discharge linkage and ACS sustainment.
Barriers to ACS Sustainment	1. Fee-for-service financing. 2. Limited methadone access and post-acute care options. 3. Interdepartmental friction and role/ownership. 4. Workforce retention and limited career pathways.

### PSAT domain 1: Environmental support (including internal partnerships)

Survey responses indicated strong perceived environmental support for ACS initiation and sustainment, particularly with respect to the presence of clinical champions and leadership support. Participants expressed somewhat less agreement regarding whether supportive administrators had adequate access to resources (see Supplementary Figure S1). Interview findings aligned with these patterns and further clarified how broad stakeholder buy-in and sustained champions across levels of the organization contributed to ACS initiation and longer-term maintenance.

#### Theme 1: Widespread stakeholder buy-in reflected shared recognition of unmet inpatient OUD Needs

Across interviews, participants described broad agreement that inpatient OUD needs were not being met and that this gap was contributing to adverse outcomes, including self-directed discharge. This shared clinical “pain point” made the rationale for an ACS intuitive to multiple stakeholder groups and facilitated early buy-in for launching services. One participant noted that frustration with repeated admissions and a lack of tools to support patients with OUD helped elevate the ACS as an institutional priority: *“Patients with substance use disorders did not have good hospital stays…had a high rate of self-directed discharge…[and] the hospital made it a priority and provided funding initially.”* (ID 12).

#### Theme 2: Leadership support and champions across levels were central to initiation and sustainment

Participants consistently described champions as critical for both starting and maintaining ACSs. Champions built coalitions across departments, negotiated workflows and staffing arrangements, and translated the clinical mission into a “business case” that resonated with leadership. One participant described that obtaining buy-in outside the behavioral health specialty required ongoing engagement with medicine and surgery, but over time the service became embedded enough that removing it would prompt resistance from clinical teams (ID 15). Leadership support was also described as necessary because ACSs are not typically revenue-generating and often require discretionary institutional investment: “*Somebody has to understand the value of this and agree to fund it.*” (ID 02).

Importantly, participants noted that sustainment depended less on one-time executive endorsement and more on dependable, mid-level champions who could advocate through routine hospital operations and demonstrate value over time. Several participants cautioned against overreliance on a single influential leader and described intentional efforts to make the ACS indispensable to reduce vulnerability to leadership turnover.

### PSAT domain 2: Funding stability

Survey results suggested that start-up resources (most commonly grants and/or departmental support) were important for launching ACSs, and that many sites transitioned over time to blended funding approaches drawing on multiple internal and external sources (see Supplementary Figure S2). Despite this diversification, interviewees frequently described funding as structurally precarious, particularly because professional billing rarely covered the full interprofessional model and because flexible discretionary support was limited. Notably, while qualitative accounts emphasized ongoing vulnerability and periodic re-justification of resources, survey respondents generally rated their current funding as stable, suggesting a distinction between day-to-day continuity and long-term security of ACS financing.

#### Theme 3: Start-up support enabled ACS launch, followed by efforts to transition to more durable institutional support

Regarding initial funding, interviewees described a range of start-up approaches, but most started with a grant or other type of support to get the ACS started. Sources of funding included state or Medicaid grants, federal grants (e.g., SAMHSA), and in some cases, institutional or departmental support. Some interviewees sought out grants to facilitate piloting or ramping up ACS services before trying to transition to relatively stable funding sources such as hospital support, partnerships with university departments, and billing for clinical services.

Despite the clear need for ACSs, a persistent challenge faced by nearly all the ACSs was assuring consistent financial support. From a purely economic standpoint, ACSs rarely generate profit and, in some instances, incur additional costs. This is partly because key team members—such as social workers and peer support specialists—are often unable to bill for their services, and because patients with OUD initiated on MOUD may experience longer hospital stays. However, interviewees described how those costs must be weighed against a range of metrics that indicate value, including lower readmission rates, improved patient health, and lower mortality rates: *“I always worry that we’re gonna get the FTE revoked. I think that my repeated pitch in terms of what we’re doing here is just the extraordinary morbidity and mortality associated with Fentanyl use and really trying to advocate this service be a necessary service as opposed to a nice thing to have for hospitals.”* (ID 20).

#### Theme 4: Funding remained structurally precarious despite diversification

Across interviews, participants described multiple pathways for launching ACSs, most commonly through time-limited start-up support (e.g., state/Medicaid or federal grants) or departmental/institutional funding that enabled early piloting and program ramp-up. Participants then described efforts to transition from these start-up mechanisms to more durable arrangements (e.g., ongoing hospital support, partnerships with university departments, and professional billing for clinician services).

However, interviewees emphasized that maintaining consistent funding remained challenging because interprofessional ACS models include essential roles that are often not billable (e.g., social work, peer support), and because MOUD initiation and care coordination may increase costs in the short term (e.g., longer length of stay). As a result, participants described having to repeatedly justify ACS value using clinical and utilization outcomes, framing the service as necessary rather than optional. One participant noted persistent concern about losing staffing support: “*I always worry that we’re gonna get the FTE revoked*,” while also emphasizing the need to communicate the scale of SUD burden and the morbidity and mortality stakes as part of sustaining the *“business case.”* (ID 2).

### PSAT domain 3: Strategic planning, organizational capacity

Survey responses reflected strong agreement that ACSs benefit from clear roles and responsibilities, ongoing efforts to increase awareness of SUD within the hospital, and proactive communication and planning for future resource needs (see Supplementary Figure S3). In contrast, respondents expressed less agreement regarding the presence of a formal sustainability plan and the adequacy of staffing levels, suggesting that while many programs had established core operational structures, longer-term planning and workforce capacity remained common areas of vulnerability.

#### Theme 5: Cross-disciplinary relationships within the hospital expanded reach and supported sustainment

Participants emphasized that sustaining an ACS required ongoing cross-departmental relationships across clinical and operational areas (e.g., pharmacy, nursing, social work, psychiatry, and specialty services such as cardiology or transplant programs). These partnerships were described as essential for solving workflow barriers, extending ACS reach to additional patient populations, and positioning the ACS as an indispensable hospital resource rather than a stand-alone consult team. As one participant noted, identifying reliable partners across units helped programs navigate operational obstacles: “*pharmacy is a huge partner…having partners in different parts like pharmacy*,* nursing*,* social work*,* medicine…knowing who’s your person*,* if you hit a wall is really important.*” (ID 06).

#### Theme 6: Interprofessional staffing and cross-departmental participation strengthened organizational capacity

Participants described staffing models that drew on multiple disciplines and, in some cases, embedded key roles (e.g., peers, social work) within existing departments to support supervision, retention, and day-to-day functioning. This cross-departmental approach was viewed as a practical strategy for sustaining roles over time, including after initial grant funding ended. One participant emphasized that embedding peer support within the social work department created the infrastructure needed for effective supervision and long-term continuity, while also highlighting the importance of demonstrating value to hospital leadership through outcomes such as readmissions and responsiveness to clinical teams.

Several sites also described using fellows and other trainees to expand clinical capacity and extend the ACS’s educational mission, which further supported sustainment by building a trained workforce pipeline. As one participant noted, incorporating trainees helped “*with the clinical work*” and “*provided an important mechanism for education and growth of the service.*” (ID 02).

### PSAT domain 4: Monitoring and evaluation

Survey responses indicated strong agreement that ACSs demonstrate value to the hospital and track outcomes or performance metrics, and that evaluation findings are used to demonstrate program success and inform planning (see Supplementary Figure S4). Respondents also endorsed the presence of productivity expectations, although agreement varied widely regarding whether explicit clinical productivity targets were in place. Overall, these findings suggest that monitoring and evaluation were viewed as central to sustainment, while expectations for productivity were less standardized across sites.

#### Theme 7: Monitoring and evaluation were viewed as essential, but measurement approaches were not required and were spontaneously developed

Participants consistently described monitoring and evaluation as central to sustaining ACSs, both to demonstrate value to leadership and to support continuous improvement of clinical workflows. At the same time, most participants indicated that hospitals did not require standardized ACS metrics, resulting in locally developed dashboards and tracking systems (see Supplementary Figure S4). Metrics varied by site but commonly included service volume (e.g., number of consultations), utilization and productivity across ACS roles, delivery of evidence-based care (e.g., MOUD initiation), and patient or provider experience. One participant described producing monthly dashboards to guide team discussions and troubleshoot workflow gaps (e.g., missed MOUD opportunities), emphasizing that this monitoring was largely self-initiated rather than mandated: “*I would create those dashboards every month…we would just present them in monthly meetings…[it] was like me randomly doing it…not…‘you must do this.*’” (ID 15).

#### Theme 8: Sites used different metrics to demonstrate value, and some commonly used hospital metrics were viewed as a poor fit

Participants described substantial cross-site variation in which metrics were prioritized to demonstrate ACS value to hospital leadership. Some emphasized outcomes such as readmissions and patient-directed discharge, while others highlighted staffing and productivity as the most salient indicators for sustainment discussions. Several participants noted that length of stay was not a useful metric for ACS impact and could be misleading, given that initiating MOUD and arranging linkage to care may extend hospitalization in the short term. One participant explained: “*We are not focusing on length of stay…we probably lengthen length of stay…[leaders care about] readmissions.*” (ID 07) Another described the recurring need to justify funding while acknowledging the complexity of using length of stay to assess the value of a consultation service.

### PSAT domain 5: External partnerships

Survey responses indicated strong perceived external partnership support, with respondents strongly endorsing that ACSs cultivate connections with community programs or leaders whose collaboration supports program goals (median 6; IQR 5–7; see Supplementary Figure S5). Interview findings expanded on these results by describing how relationships with outpatient treatment programs, harm reduction services, and post-acute care settings (including skilled nursing facilities) were integral to discharge planning, linkage to ongoing care, and longer-term ACS sustainment.

#### Theme 9: External partnerships were essential for post-discharge linkage and ACS sustainment

Participants consistently emphasized that ACS effectiveness and sustainment depended on strong relationships with community partners, including outpatient treatment programs, harm reduction services, and post-acute care settings such as skilled nursing facilities (SNFs). These partnerships were described as critical for timely linkage to ongoing care after discharge and, in some cases, for preventing refusals of patients initiated on MOUD in the hospital. Participants also described investing substantial effort in cultivating and maintaining these connections, often through regular communication and shared workflows. One participant underscored that outpatient linkage capacity was foundational to ACS success: *“The number one facilitator…is our strong outpatient programs that we can link people to…if you don’t have that you can’t do anything.*” (ID 08) Others described more formalized collaboration, including involving community program leaders in regular meetings and developing fast tracking processes to expedite referrals from the ACS to outpatient services.

### Cross-cutting barriers to ACS sustainment

In addition to PSAT domain facilitators, participants described several persistent barriers that threatened sustainment across settings. These barriers largely reflected structural financing constraints, gaps in community treatment capacity, and organizational/operational challenges within hospitals. We describe the most common barriers below.

#### Barrier 1: Fee-for-service financing

Participants emphasized that fee-for-service reimbursement typically covered only prescribing clinician time, leaving core non-billable functions—care coordination, monitoring, peer support, and social work—dependent on discretionary institutional support or time-limited grants. This financing structure was viewed as misaligned with the preventive and system-level value of ACSs (e.g., reducing overdose risk and avoidable utilization) and made expansion of non-billable roles difficult. As one participant stated that “*fee-for-service is not really how we show the most benefit.*” (ID 08) Participants suggested that the ACS value proposition may align better with alternative payment models than with volume-based reimbursement.

#### Barrier 2: Limited methadone access and post-acute care options

A subset of participants described insufficient community methadone capacity and access to post-acute placement as major threats to sustained impact. Participants noted that SNFs and sub-acute rehabilitation facilities may refuse patients receiving methadone or other MOUD, creating discharge bottlenecks and disrupting continuity of care. One participant highlighted the challenge of securing appropriate placement for non-ambulatory patients initiated on methadone, describing persistent reluctance among nursing facilities to accept patients with substance use histories.

#### Barrier 3: Interdepartmental friction and role/ownership

Nearly one-third of participants described tension between departments—most commonly medicine/hospitalist services and psychiatry—as an impediment to sustainment. Participants attributed these challenges to disagreements about where the ACS belongs within the hospital, differences in professional culture and workforce availability (e.g., relative interest in consult work), and underlying differences in philosophy regarding inpatient treatment of substance use disorders. One participant described the operational complexity of running a service across disciplines in a siloed world and the resulting pressure to restructure service ownership and staffing. Another described delayed collaboration due to perceived competition and differences in beliefs about treating substance use disorders in the hospital setting.

#### Barrier 4: Workforce retention and limited career pathways

Participants also described workforce challenges, including recruiting and retaining staff with appropriate experience, turnover, and limited advancement pathways—particularly for advanced practice providers (APPs) and peer recovery roles. These challenges were compounded by the evolving status of peer roles within hospital systems and, in some cases, leadership turnover and limited familiarity with addiction services among administrators. One participant emphasized the need for clearer promotion pathways and support structures for APPs and peers, noting that these roles may be undervalued within traditional health care career ladders.

## Discussion

While results from the START RCT have demonstrated the effectiveness of ACSs in increasing initiation of medications for OUD and improving linkage to treatment after discharge, and other studies have also demonstrated effectiveness of ACSs on these and other critical patient outcomes, less is known about the organizational and contextual factors that enable ACSs to be successfully launched and sustained within hospitals [5–7]. Our mixed-methods analysis addresses this gap by identifying key facilitators of and barriers to ACS implementation and sustainment.

Our findings highlight the importance of environmental support, including stakeholder buy-in, leadership endorsement, and broad recognition of the clinical need for OUD care, as a foundational condition for success. Internal champions, particularly those in mid-level leadership roles, are pivotal for building coalitions, securing resources, and aligning ACS initiatives with hospital priorities. While startup funding often comes from soft funds, successful ACSs tend to evolve toward a blended funding model, with many programs relying on staffing or indirect subsidization by teaching services. Cross-departmental integration and interprofessional staffing can potentially support sustainability and help expand the ACSs’ reach by embedding practices into existing hospital workflows. External partnerships with outpatient programs and community organizations are thought to be critical for enabling successful discharge plans and reinforcing the ACS’s role within a broader continuum of care.

Although evaluation practices differed across hospitals and typically are not required, many ACSs use performance monitoring to strategically demonstrate their value, such as tracking readmission rates for patients who were seen by the ACS and sharing these data with leadership to justify continued investment. Ongoing monitoring, even if informal, is considered a critical component of sustainability.

Persistent barriers to ACS sustainability suggested by our findings include fee-for-service reimbursement shortfalls, lack of access to methadone services in many communities, difficulties transferring patients on MOUD to SNFs, interdepartmental coordination challenges, and workforce retention issues. Despite relatively high PSAT domain scores, indicating strong internal support for sustainment, many ACSs in this study reported continuing to struggle with financial vulnerability. Echoing findings from Priest et al., interviewees described a fundamental misalignment between ACS functions and prevailing reimbursement models that primarily support prescribers but often exclude other essential team members, such as social workers and peer specialists [12]. Finally, findings from this study caution against overreliance on individual champions, emphasizing instead the need for institutional structures and policies to promote long-term program stability beyond the tenure of specific leaders.

In Table 4, we summarize key takeaway lessons that practitioners (e.g., those involved with starting or sustaining an ACS) that might be useful throughout the development and evolution of their ACS. While each ACS ultimately needs to be tailored to its local environment, these general takeaways might be informative for those operating in a wide range of hospital environments.

**Table 4:** Summary of takeaways related to ACS sustainment

PSAT Domain	Key Takeaways for Practitioners
Domain 1: Environmental Support (Including internal partnerships)	Facilitator:
	Stakeholder buy-in
	Broad recognition of the clinical need for OUD
	Leadership support
	Champions throughout the hospital
Domain 2: Funding Stability	Facilitator:
	Grant or other support for start-up phase
	Transition to a stable funding source including internal funding
	Barrier:
	ACSs typically do not earn a profit due to key staff members who cannot bill
Domain 3. Strategic Planning, Organizational Capacity	Facilitator:
	Cross-disciplinary relationships within the hospital
	Interprofessional staffing mix and cross-departmental participation
Domain 4. Monitoring and Evaluation	Facilitator:
	Monitoring and evaluating the ACS are considered crucial to sustainment
	Barrier:
	Metrics are not standardized or formalized within hospitals or across sites
Domain 5. External Partnerships	Facilitator:
	Relationships with community partners help ensure linkage from the hospital to ongoing care
Ongoing Barriers to ACS Sustainment	Barriers:
	Fee-for-service funding model is challenging, especially outside of academic settings
	Lack of adequate methadone treatment services outside the hospital, and barriers to linking patients on MOUD to acute care and skilled nursing facilities (SNFs), impede referral and linkage to post-discharge care.
	Friction between hospital departments can impede ACS service delivery
	Workforce issues, such as finding staff to cover the ACS and staff turnover, are challenges for some ACSs

This study faces some limitations including that the ACSs represented in this study generally are located within relatively well-resourced hospitals, many with secondary affiliations (e.g., with universities or with the VA) and many are in urban areas. Priest et al. noted a similar limitation in their 2020 study [12]. It seems likely that well-resourced ACSs are more likely to be initiated, sustained, and better known to those in the addiction medicine field. Future research could explore the extent to which less well-resourced hospitals have tried or plan to try implementing ACSs. Further, our study did not include perspectives from bedside nurses, social workers, pharmacists, patient navigators, and other frontline staff who may serve on ACS teams and/or work alongside ACSs, and who may therefore shape implementation processes and sustainment through their roles in care coordination and hospital workflow integration. Future research could also explore cases in which ACSs were initiated but not sustained over time. Finally, while there was some variation in the taxonomic category of the ACSs in this study, interview themes tended to be consistent across participants. Further research can build on Englander et al.’s [11] call to explore outcomes of different types of ACS models by exploring nuances in implementation and sustainability in relation to ACS outcomes.

## Conclusion

In this study of factors related to sustaining ACSs, critical findings include the importance of stakeholder buy-in, leadership support, and champions at different levels; the use of blended funding sources; the importance of cross-departmental collaboration; the value of metrics to monitor ACSs; and the role of community linkages in sustaining ACSs. Understanding potential sustainability of ACS within PSAT domains may be of use to providers and administrators for planning, implementing, and supporting ACSs over time.

## Supplementary Information

Below is the link to the electronic supplementary material.

Supplementary Material 1

Supplementary Material 2

## Data Availability

Data from the survey and deidentified qualitative data generated and analyzed during this study can be made available from the corresponding author on reasonable request and with execution of appropriate Data Use Agreements.

## Footnotes

1 We do not report the extent to which ACSs in our sample overlap (or do not overlap) with ACS samples from other articles, including Englander et al. (2022) in order to protect the confidentiality of participants.

## Abbreviations

ACS: Addiction Consult Service / Addiction Consultation Service
AMA: Against Medical Advice
APP: Advanced Practice Provider
CI: Confidence Interval
CMO: Chief Medical Officer
FTE: Full-Time Equivalent
IQR: Interquartile Range
IRR: Inter-Rater Reliability
MOUD: Medication for Opioid Use Disorder
OUD: Opioid Use Disorder
PSAT: Program Sustainability Assessment Tool
RCT: Randomized Clinical Trial
SNF: Skilled Nursing Facility
START: Substance Use Treatment and Recovery Team
SUD: Substance Use Disorder
U.S.: United States
VA: Veterans Affairs

## References

[1] Substance Abuse and Mental Health Services Administration (SAMHSA). Key substance use and mental health indicators in the United States: results from the 2024 National Survey on Drug Use and Health. Rockville (MD): Center for Behavioral Health Statistics and Quality, SAMHSA; 2025.

[2] Wakeman S, Herman G, Wilens T, Regan S. The prevalence of unhealthy alcohol and drug use among inpatients in a general hospital. Subst Abus. 2020;41(3):331–9. 10.1080/08897077.2019.1635961 31368860

[3] Rosen AD, Takada S, Juillard C, Fernandez Montero YL, Richards AM, Ngekeng S, et al. Unpacking the link between substance use disorders and 30-day unplanned readmission. Addiction. 2025. 10.1111/add.70136 PMC12586751 40698401

[4] Lewer D, Eastwood B, White M, Brothers TD, McCusker M, Copeland C, et al. Fatal opioid overdoses during and shortly after hospital admissions in England: A case-crossover study. PLoS Med. 2021;18(10):e1003759. 10.1371/journal.pmed.1003759 PMC8491890 34610017

[5] Ober AJ, Murray-Krezan C, Page K, Friedmann PD, Anderson J, Osilla KC, et al. Hospital Addiction Consultation Service and Opioid Use Disorder Treatment: The START Randomized Clinical Trial. JAMA Intern Med. 2025;185(6):624–33. 10.1001/jamainternmed.2024.8586 PMC11976642 40193131

[6] Ober AJ, Osilla KC, Klein DJ, Burgette LF, Leamon I, Mazer MW, et al. Pilot randomized controlled trial of a hospital-based substance use treatment and recovery team (START) to improve initiation of medication for alcohol or opioid use disorder and linkage to follow-up care. J Subst Use Addict Treat. 2023;150:209063. 10.1016/j.josat.2023.209063 PMC10330512 37156424

[7] McNeely J, Wang SS, Rostam Abadi Y, Barron C, Billings J, Tarpey T, et al. Addiction consultation services for opioid use disorder treatment initiation and engagement: a randomized clinical trial. JAMA Intern Med. 2024. 10.1001/jamainternmed.2024.3422 PMC11287446 39073796

[8] Danovitch I, Korouri S, Kaur H, Messineo G, Nuckols T, Ishak WW, et al. The addiction consultation service for hospitalized patients with substance use disorder: An integrative review of the evidence. J Subst Use Addict Treat. 2024;163:209377. 10.1016/j.josat.2024.209377 38657952

[9] Trowbridge P, Weinstein ZM, Kerensky T, Roy P, Regan D, Samet JH, et al. Addiction consultation services – Linking hospitalized patients to outpatient addiction treatment. J Subst Abuse Treat. 2017;79:1–5. 10.1016/j.jsat.2017.05.007 PMC6035788 28673521

[10] Weinstein ZM, Wakeman SE, Nolan S. Inpatient Addiction Consult Service: Expertise for Hospitalized Patients with Complex Addiction Problems. Med Clin North Am. 2018;102(4):587–601. 10.1016/j.mcna.2018.03.001 PMC6750950 29933817

[11] Englander H, Jones A, Krawczyk N, Patten A, Roberts T, Korthuis PT, et al. A Taxonomy of Hospital-Based Addiction Care Models: A Scoping Review and Key Informant Interviews. J Genl Inter Med. 2022;37(11):2821–33. 10.1007/s11606-022-07618-x PMC9411356 35534663

[12] Priest KC, Englander H, McCarty D. Now hospital leaders are paying attention: A qualitative study of internal and external factors influencing addiction consult services. J Subst Abuse Treat. 2020;110:59–65. 10.1016/j.jsat.2019.12.003 PMC7181883 31952629

[13] Luke DA, Calhoun A, Robichaux CB, Elliott MB, Moreland-Russell S. The Program Sustainability Assessment Tool: A New Instrument for Public Health Programs. Prev Chronic Dis. 2014;11:E12. 10.5888/pcd11.130184 PMC3900326 24456645

[14] Dedoose, Version. 10.0.25, cloud application for managing, analyzing, and presenting qualitative and mixed method research data Los. Angeles, CA: SocioCultural Research Consultants, LLC; 2023.

[15] Krippendorff K, Bock MA. The content analysis reader: Sage Publications, Inc; 2008.

[16] Braun V, Clarke V. Using thematic analysis in psychology. Qualitative Res Psychol. 2006;3(2):77–101.

[17] Wickham H. ggplot2: Elegant graphics for data analysis. New York: Springer-; 2016.

