# Supplementary material for: Implementation and sustainability of hospital-based addiction consultation services
Source: Addict Sci Clin Pract. 2026 Jul 10;21:72. doi: 10.1186/s13722-026-00695-x (PMC13647775; doi:10.1186/s13722-026-00695-x)
Supplement: Supplementary file 1 [file 13722_2026_695_MOESM1_ESM.docx]

**START Implementation and Sustainability Article**

**Supplementary Figures: S1-S5**

**Figure S1.** Median and IQR results for survey items assessing environmental support. High median scores (above 5 on a scale from 1 to 7) indicate strong support for the three items.

**Figure S2.** Median and IQR results for survey items assessing funding stability. The mixed median scores indicate a range of views related to funding stability.

**Figure S3.** Median and IQR results for survey items assessing strategic planning and organizational capacity. While there is some variance, the mostly high median scores (above 5 on a scale from 1 to 7) indicate support for the items addressing strategic planning and organizational capacity.

**Figure S4.** Median and IQR results for survey items assessing monitoring and evaluation. Median scores show some variance. Higher median scores (above 5 on a scale from 1 to 7) indicate relatively more support for two of the five items.

**Figure S5.** Median and IQR results for PSAT domain items assessing external partnerships. The high median score (above 5 on a scale from 1 to 7) indicates support for this item.
