# Supplementary material for: Implementation and sustainability of hospital-based addiction consultation services
Source: Addict Sci Clin Pract. 2026 Jul 10;21:72. doi: 10.1186/s13722-026-00695-x (PMC13647775; doi:10.1186/s13722-026-00695-x)
Supplement: Supplementary file 2 [file 13722_2026_695_MOESM2_ESM.docx]

**START Implementation and Sustainability Article**

**Supplementary Tables: S1 and S2**

**Table S1. Interview Guide**

| **PSAT category** | **Interview Questions** |
| --- | --- |
| Environmental support | - Who were the key decision-makers at [HOSPITAL] who influenced whether the ACS was adopted and sustained? - What partnerships within the hospital have been critical to implementing and sustaining the ACS? |
| Funding stability | - How did funding work during the initiation and development of the ACS? - How does funding work for sustaining the ACS? - Does the ACS bill for its services? - What mechanisms or sources fund this program? How is funding sustained? - What have been the biggest funding barriers? How have these been overcome? |
| Organizational capacity | - You told us previously [...]. Were these the roles you started with? If not, how have the roles changed and why? - Has the presence of the ACS altered or changed other clinical processes or services? |
| Strategic planning | - How does strategic planning for new programs typically occur at [HOSPITAL]? - Who makes sure that planning for programs aligns with existing hospital goals? - What implementation strategies are typically used to implement new practices? - What do you think the most important factor was for implementing this ACS? |
| Communications | - Tell me about how you shared information about the ACS in the hospital when it was first implemented? - How does communication with those hospital stakeholders work today? - To what extent have community stakeholders been informed about the ACS? |
| Monitoring  and Evaluation | - How is the ACS monitored and evaluated? - Who oversees monitoring and evaluation? - What metrics or goals does the ACS report on? - Has the ACS evolved over time? Is the ACS regularly adapted to meet specific objectives? - With whom do you communicate successes related to the ACS? |
| External Partnerships | - What partnerships outside of the hospital have been critical to implementing and sustaining the ACS? - What has been the role of these partners in implementing the ACS? |
| Barriers | - What do you think were the most significant barriers to implementing and sustaining the ACS at [HOSPITAL]? - Do any of these barriers still exist? Can you discuss any barriers impacting the ACS now? |

**Table S2. Codes and Definitions for Analysis of Interview Data**

| **Codes** | **Definition** |
| --- | --- |
| Establishment of the ACS (Driving Factors/How and why did you start). | Role of champions or advocates or specific leaders; Extent to which the perceived need drove the start of the ACS; Hospital needs that helped facilitate the start of the ACE; Extent to which external factors like policy helped facilitate the start of the ACS (Financial topics should be coded under IE. Initial Funding)  Child codes:   - Champion(s) - High need/hospital reasons - Policy/Medicare mandate - other |
| Decisionmakers/ leadership/championship | Influence or role of leaders/decisionmakers on sustainment of ACS |
| Location in hospital | Discussion about issues related to location of ACS in hospital |
| Communications | Communication/relationships w/in the hospital; Community/linkage partnerships  Child codes:   - Communication/relationships w/in the hospital   Communication/relationships with community/linkage partnerships |
| Staffing | Staffing composition plus issues related to staffing composition or needs; why and how the ACS evolved over time |
| Funding | Types of funding used to support the ACS or related funding issues |
| Activities, services provided, workflow integration & coordination | What the ACS does / how the ACS provides its services, how activities have evolved |
| Tracking/monitoring/ evaluation | How the ACS is or is not monitored, evaluated, or tracked; oversight of ACS |
| Barriers/challenges to implementation and sustainment | Barriers or challenges that impact the current ACS |
| Facilitators to implementation and sustainment | Facilitators that impact the current ACS |
| Needed ACS improvements/deficits/ challenges/future plans & changes | Discussion about needed ACS improvements/deficits/challenges/future plans & changes |
